# Treatment of Dysmenorrhea by Quinine and Prussiate of Iron

**Published:** 1850-08

**Authors:** H. A. Bignon

**Affiliations:** Augusta, Ga.


*Treatment of Dysmenorrhea by Quinine and Prussiate of Iron.* By H. A. Bignon, M. D., Augusta, Ga.—The frequent occurrence of dysmenorrhea, and its painful and intractable character, render it a subject of deep interest to the physician. As the most approved modes of treatment often effect little more than a slight palliation, I am induced to report a few cases treated with quinine and prussiate of iron, in the hope that others may be induced to test these remedies in similar cases.

Case I.—About the first of July, 1848, I was called to see Celia, a negro woman about 28 years of age, and of very delicate constitution: she was then laboring under dysmenorrhea, and on inquiry I found that she had been affected with it for some nine years, during which time she had been under the treatment of several physicians, and as far as I could learn, had been put on the use of purgatives, tinct. of guaiacum, and all the usual remedies, with only slight relief, if any. I immediately prescribed a warm hip bath with mustard, and ten grains of Dover's powder, under the influence of which she was not long in falling to sleep, and got a good night's rest. On my visit the next day, I found that the pains had returned, and she was suffering very much. I suspected the existence of a clot, from the character of the pains (resembling those of labor) and immediately gave her a teaspoonful of the wine of ergot, and repeated the dose about every ten minutes, until she had taken three spoonsful, after which she passed a clot about the size and shape of an almond, to the entire relief of all her suffering. I prescribed another hip bath and ten grs. of Dover's powder for the night, and left her.

I did not see my patient again until about two weeks after, when she came to see if I could not give her something to prevent her suffering so much at the next approach of the catamenia. I put her on the use of pills, consisting of three grains of quinine and three grains of prussiate of iron, of which she was to take one three times a-day, and gave her a mixture of camphor (Dewees) to take in case she suffered much at the next period, and did not see her again until about one week after she had had a return of the discharge (making about three weeks since my last interview with her,) when she came to get more of the pills, saying that she thought they had helped her a good deal, but not cured her. I made another box of the pills, and kept her on the use of them for the space of about six months, when she came to me quite another looking woman, and entirely free from the disease. I have seen her frequently since, and she continues well.

CASE II.—Maria, a negro woman, aged about 32 years, and of slight frame, applied to me in the month of September, 1848, for the relief of dysmenorrhea, which she had had since a cold caught after confinement seven years previous. As the case was very similar to the previous one, I will not go into a detail of it, but merely say that I put her upon the same treatment, and in the space of about seven months I had the gratification of seeing her quite well again.

CASE III.—Ann, a negro woman, aged about 20 years, well made and of large stature, applied to me in the month of November, 1849, for the relief of dysmenorrhea. The case was similar to the others, excepting that Ann was then nursing a child of three years of age, and also complained more of pain in the back than did the others. I put her upon the same treatment as the other cases, with the addition of a blister to the sacrum, and made her wean the child. This case is under my care at the preseet time, and at the last period she says that the discharge was quite natural, and that the pain was scarcely to be felt.—*South. Med. and Surg. Jour.*

